# Niche partitioning and trait tradeoff strategies enable plants to coexist under interspecific competition in restored wetlands

**DOI:** 10.3389/fpls.2025.1539136

**Published:** 2025-04-02

**Authors:** Shenglin Yang, Zhen Yuan, Bibi Ye, Feng Zhu, Xiaoxian Tang, Rui Gao, Zhaosheng Chu, Xiaowei Liu

**Affiliations:** ^1^ State Key Laboratory of Environmental Criteria and Risk Assessment, National Engineering Laboratory for Lake Pollution Control and Ecological Restoration, State Environmental Protection Key Laboratory for Lake Pollution Control, State Environmental Protection Key Laboratory of Drinking Water Source Protection, Chinese Research Academy of Environmental Sciences, Beijing, China; ^2^ College of Water Science, Beijing Normal University, Beijing, China; ^3^ Institute of Lake Ecology and Environment, Anhui Provincial Lake Chaohu Administration, Hefei, China; ^4^ School of Biology, Food, and Environment, Hefei University, Hefei, China

**Keywords:** interspecific competition, coexisting strategies, niche partitioning, traits tradeoff, vascular plants

## Abstract

**Background:**

Niche partition and traits tradeoff theory were primary strategies for plants coexistence. However, specific strategies of plants remained to be verified to guide community configuration and biodiversity maintenance in ecological restoration.

**Methods:**

The variation of plants composition and niche breath were utilized to examine the temporal and spatial niche partition strategies, respectively. Meanwhile, the chi-square (χ^2^), Spearman rank correlation coefficient (r_ij_), Ochiai index (OI) were employed to analyze the interspecific relationship of 30 predominant species from species pool of 220 vascular plants. Besides, the Lotka-Volterra model was utilized to reveal the traits tradeoff strategies of predominant species from five vegetation formations.

**Results:**

About 62.41% pairs of wetland species were niche partitioned while 37.58% of species pairs were niche overlapped. In temporal scale, 60.5% of species occurred either in spring or autumn while 39.5% occurred in both seasons. Meanwhile, significant change of relative height (RH) and relative coverage (RC) were observed in constructive species and auxiliary species. Height tradeoff strategy ( $\frac{\Delta RH}{\Delta RC}>1$ ), coverage enlarge strategy ( $\frac{\Delta RH}{\Delta RC}<1$ ), or both strategies ( $\frac{\Delta RH}{\Delta RC}=1$ ) observed in wetland plants.

**Discussion:**

Our finding testified that the temporal niche partition and traits tradeoff strategies are objectively observable in wetland plants. These findings on coexistence strategies can be used in the configuration of plants communities and the biological control of alien invasive plants.

## 1 Introduction

Restored or created wetlands have been established globally to promote biodiversity conservation, but often end up with undesirable outcomes due to unpredictable ecological succession trajectories in changing environments (Baumane et al., 2021; Funk and Wolf, 2016; Jenkins et al., 2021; Salaria et al., 2018). For these factors such as biological invasions, unreasonable configuration and interspecific competition between native and non-native species can lead to the exclusion of less competitive species (Adler et al., 2018; Carboni et al., 2021; Tilman, 1990). The exclusion of species is mainly attributed to the competition for resources such as light, nutrient, and water (Silvertown, 2004). Traditional theories about the mechanisms of species coexistence include environmental heterogeneity, niche and resource partitioning (Adler et al., 2013; Aguiar et al., 2001; Fargione and Tilman, 2005; Gaudet and Keddy, 1988; Meilhac et al., 2019). The partition of different species depended on fluctuation of plants population densities and resource in space and time (Chesson, 2000). Besides, the phenology as the avenue of niche partition supplemented the high biodiversity of wetlands (Levine et al., 2022; Pak et al., 2023). Although the mechanisms for plants coexistence had been developed, the processes and strategies of plants in managing interspecific competition among heterospecific species remained unclear.

Previous studies assumed that coexistence functioned in two ways: the decrease of fitness difference or the increase of negative intraspecific interactions (Chesson, 2000). The fitness of plants could be revealed by its functional traits which were highly correlated with its competitive ability (Gaudet and Keddy, 1988). These traits of plants including canopy height, leaf size, growth rate, plant lifespan, aboveground and belowground biomass that could be employed to assess plant competitiveness (Adler et al., 2013; Grime, 1974; Lhotsky et al., 2016; Puglielli et al., 2024). In natural wetlands, the environmental heterogeneity acted as environmental filter that leaded to the convergence of plants traits between native and exotic species (Cleland et al., 2011). On the contrary, niche-based community theory predicted that competitive exclusion contributed to the divergence of traits between species pools (Cleland et al., 2011). Trait-based community assembly theory suggested that plants functional traits could be used to reveal the biotic and abiotic processes of plant coexistence (de Bello et al., 2012).

But there remained challenges in how to quantify the variance of trait-based characters. In stabilizing process, species inclined to offset disadvantages or enhance certain traits to protect themselves from being excluded by intraspecific competition (Adler et al., 2013; Chesson, 2000; Leibold et al., 2022). The trait-based tradeoff strategies of plant can provide insights into plant behaviors when competing with neighbor species (Cleland et al., 2011; de Bello et al., 2013; Kempel et al., 2011; Puglielli et al., 2024). Additionally, mathematical methods like interspecific correlation index and Lotka–Volterra competition models had been developed to quantify the competition between heterospecific species (Broekman et al., 2019; Chesson, 2000). However, the variables in the Lotka-Volterra competition model were morphologically, physiologically and behaviorally unrealistic (Tilman, 1990). Thus, simplified and easy measured variables are urgently needed to explain the mechanism underlying plant coexisting.

Light, as an essential resource for plant photosynthesis, tended to be the primary limiting factor for plant coexistence when nutrient supply was relatively constant in the same macrohabitat (Eskelinen et al., 2022). The functional traits of plants are primarily linked to their competitive abilities for light (Gaudet and Keddy, 1988). As plant competitiveness for light and soil space is often size-asymmetric, morphological traits can be used to understand plants’ strategies (Aschehoug et al., 2016; Craine et al., 2013; Öpik et al., 2013). For example, the alien invasive plant species (*Solidago canadensis, Bidens frondose* and *Erigeron canadensis*) obtained greater phenological plasticity like phenology and reproduction than native species (Cao et al., 2018; Ren et al., 2023). Thus, morphological parameters such as plants’ phenology, relative height, and coverage can be utilized as proxies to quantify the variation plants traits under interspecific competition (Aschehoug et al., 2016).

To deepen our understanding of the impacts of interspecific competition on plant diversity and coexistence, empirical studies on plants coexisting strategies are necessary. This involves analyzing pairwise interactions between native and non-native species within regional species pools among 19 lakeside wetlands. Additionally, spatial and temporal niches, along with phylogenetic information, are studied to uncover niche partitioning strategies. Morphological metrics are also incorporated into Lotka–Volterra competition models to visualize the tradeoff strategies of plants in competitive environments. The results of this study can provide a theoretical basis in the guide of plants conservation in restored or created wetlands.

## 2 Materials and methods

### 2.1 Study regions

**Figure 1:**
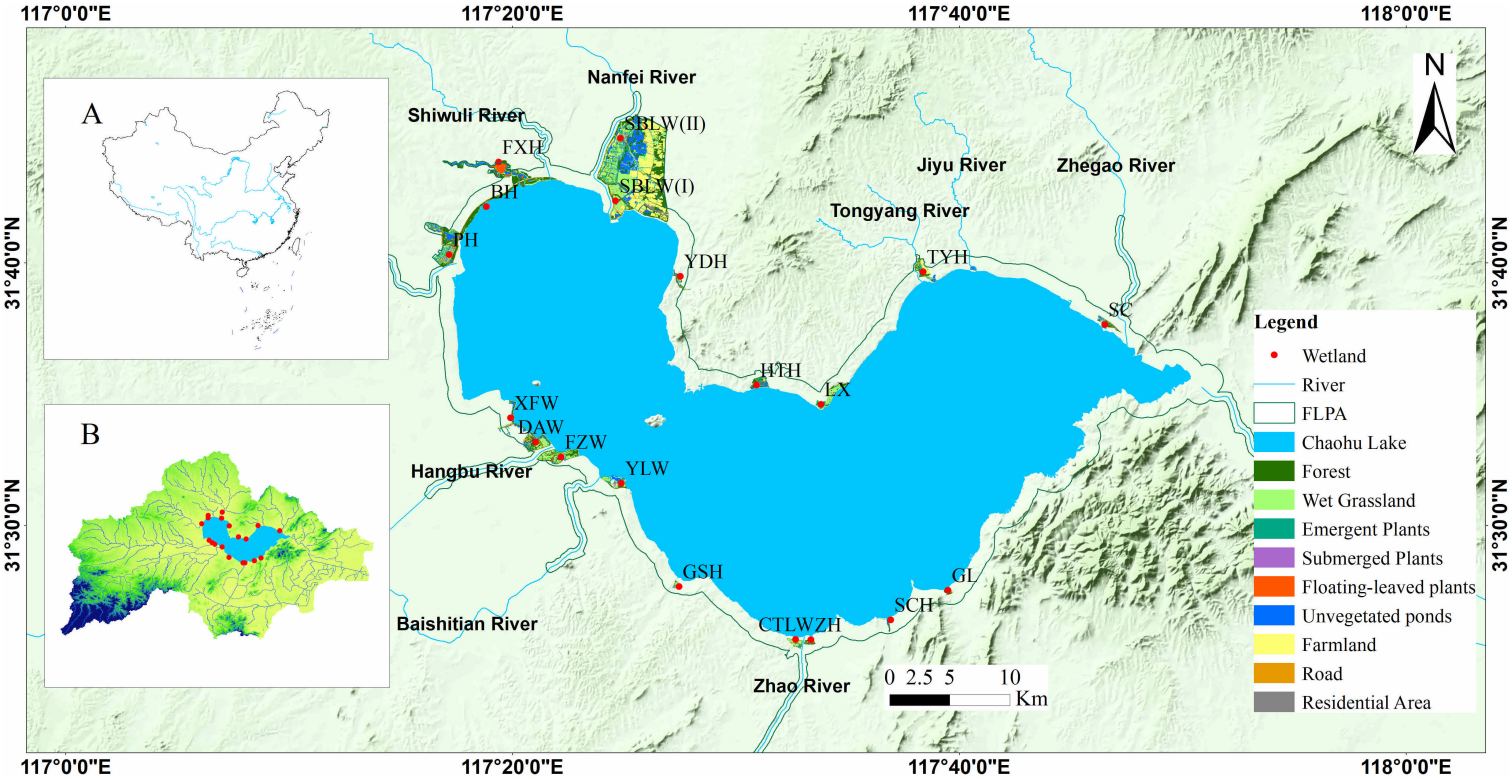
Location of studied 19 restored wetlands in the lakeside of Chaohu Lake basin. **(A, B)** Geographical position of Chaohu Lake Basin.

This study focused on plants diversity and stability of aquatic communities in 19 restored lakeside wetlands of Chaohu Lake which is the fifth largest freshwater lake in China (Chen et al., 2023). The studied 19 restored lakeside wetlands located in the First Level Protected Area (FLPA) with a scope of 1 kilometer extended the shoreline of Chaohu Lake. The completed date of the 19 restored wetlands was listed in **Supplementary Table S1**. This region experiences a subtropical monsoon climate, with an annual precipitation of 1,124.4 mm and a temperature of 16.7°C (Yang et al., 2024). Meanwhile, the studied 19 restored lakeside wetlands encompassed a variety of microhabitats such as plains, hills, marshes, and ponds (**Figure 1**). The combination of diverse geological conditions and suitable climate in this area facilitated the colonization of abundant vascular plants. According to the lifeform and ecological types of vascular plants, lakeside wetlands vegetation was classified into five vegetation formations, including upland plants, wet grasslands, emergent plants, floating-leaved plants, and submerged plants (Jenkins et al., 2021; Yang et al., 2024).

### 2.2 Data sources

To reveal plants coexisting strategies, a number of 19 sampling transects were conducted among 19 lakeside wetlands of Chaohu Lake in the spring (April 20th to May 12th) and autumn (September 23rd to October 12th) of 2023. Each sampling-transect of restored wetland traversed five vegetation formation with a length ranged from 0.5 to 2 kilometers that was positively correlated with the width of wetland. Meanwhile, a number of 8-15 herbaceous plant quadrats (1×1m) were set at each transect and a total of 427 samples were conducted in this study. Plant characteristics including abundance, height, coverage, phenology, above ground biomass of plants were recorded to analyze the spatial niche and interspecific competition among vascular plants (Yang et al., 2024). Additionally, 10 traits encompassing functional, morphological, and phenological parameters of plants were examined to elucidate the competitive strategies of plants (Wang et al., 2012). Specifically, the content of the 10 traits were listed in **Supplementary Table S2**. The data of morphological, and phenological parameters of vascular plants were collected from field observation and *Flora of China* (Wu and Zhou, 2001).

### 2.3 Data analysis

The chi-square (*χ^2^*) statistics were utilized to examine the interspecific associations of functional groups (Hurlbert, 1969). The *χ^2^* value was calculated according to the following formula (Gu et al., 2017; Jin et al., 2022).


(1) $$\chi^{2}=\frac{{\left(|ad-bc|-0.5\text{N}\right)}^{2}\text{N}}{\left(a+b)(a+c)(b+d)(c+d\right)}$$

Where a represents the number of quadrats where both species *i* and *j* occurred. b represents the number of quadrats where only species *i* occurred. c represents the number of quadrats where only species *j* occurred. d represents the number of quadrats where neither species *i* nor *j* occurred, and N represents the total number of quadrats. When χ^2^ < 3.841, there is no interspecific association between species; when 3.84 ≤χ^2^ ≤ 6.635, there is a moderate association between species; and when χ^2^ > 6.635, there is significant association between species (Gu et al., 2017).

The Ochiai index (OI) was used to assess the degree of isolation between species. The OI index was calculated according to the following equation (Hu et al., 2022).


(2) $$OI=\frac{a}{\sqrt{\left(a+b)(a+c\right)}}$$

Where the value of a, b, and c in Equation 2 are the same as those in Equation 1. When OI value is 0, indicating that the species are completely independent. The closer the OI value is to 1 and χ^2^>0, the higher the probability of two species cooccur in the same habitat.

The Spearman rank correlation coefficient (r_ij_) was used to assess the level of linear correlation between independent species (Gauthier, 2001). The calculation of r_ij_ was based on the following equations:


(3) $$r_{ij}=\frac{\sum_{k=1}^{N}(x_{ik}-\bar{x_{i}})(x_{jk}-\bar{x_{j}})}{\sqrt{\sum_{k=1}^{N}{\left(x_{ik}-\bar{x_{i}}\right)}^{2}\sum_{k=1}^{N}{\left(x_{jk}-\bar{x_{j}}\right)}^{2}}}$$

(4) $$r_{\left(i,j\right)}=1-\frac{6\sum_{k=1}^{N}d_{k}^{2}}{N^{3}-N}$$

(5) $$d_{k}=X_{ik}-X_{jk}$$

Where $r_{\left(i,j\right)}$ is the rank correlation analysis between species *i* and *j*. *N* is the total number of quadrats. $X_{ik}$ and $X_{jk}$ are the rank vector of *i* and *j* that were converted from the quantity of species *i* and *j* in *k*th quadrat. dk is the difference of the rank vector of species *i* and *j*. The value of *r_(i,j)_* is ranged from -1 to 1. If *r_(i,j)_* > 0, the species have a positive correlation, and if *r_(i,j)_* < 0, species have a negative correlation.

The niche breadth (B) was utilized to analyze the niche of plants (Colwell and Futuyma, 1971; Jin et al., 2022).


(6) $$B=\frac{1}{\sum P_{j}^{2}}=\frac{N_{T}^{2}}{\sum N_{j}^{2}}$$

The N_T_ is the total number of species *i* among five vegetations formations while *N_j_* is the number of species *i* in each vegetation formation.

Morphological traits of vascular plants, such as relative average height (RH), relative coverage (RC), and relative abundance (RA), were used to assess plant competitiveness. The important value (IV), calculated as the average of relative abundance (RA), relative frequency (RF), and relative coverage (RC), was used to determine species significance in wetland ecosystems. Dominance (D) was employed to indicate plant prevalence among numerous species. These traits were calculated as the following equations (Gu et al., 2017; Jin et al., 2022).


(7) $$RH=H_{i}/\sum_{i=1}^{S}H_{i}\times 100\%$$

(8) $$RC=C_{i}/\sum_{i=1}^{S}C_{i}\times 100\%$$

(9) $$RA=A_{i}/\sum_{i=1}^{S}A_{i}\times 100\%$$

(10) $$RF=F_{i}/\sum_{i=1}^{S}F_{i}\times 100\%$$

(11) $$RW=W_{i}/\sum_{i=1}^{S}W_{i}\times 100\%$$

(12) IV=(RA+RC+RF)/3

(13) D=(RH+RC+RA+RF+RW)/5

The height of individuals of the *i*th species is represented by H_i_. C_i_ signifies the total coverage of the *i*th species, while A_i_ represents the total number of individuals of the *i*th species. *S* indicates the total number of species in the quadrat, and F_i_ denotes the total number of quadrats in which the *i*th species appears.

The variation of relative height (Δ*RH*) and relative coverage (Δ*RC*) were used to evaluate the growth rate of plants. The calculation of ΔRH and ΔRC was based on the following equations:


(14) $$\Delta RH=RH_{t2}-RH_{t1}$$

(15) $$\Delta RC=RC_{t2}-RC_{t1}$$

Where $RH_{t2}$ is the relative height of plants at time t_2_. $RH_{t1}$ is the relative height of plants at time t_1_. Analogously, $RC_{t2}$ is the relative coverage of plants at time t_2_ while $RC_{t1}$ is the relative coverage of plants at time t_1_.

All the data were calculated with Excel and analyzed via Origin 2024 pro.

## 3 Results

### 3.1 Species pool in this region

**Figure 2:**
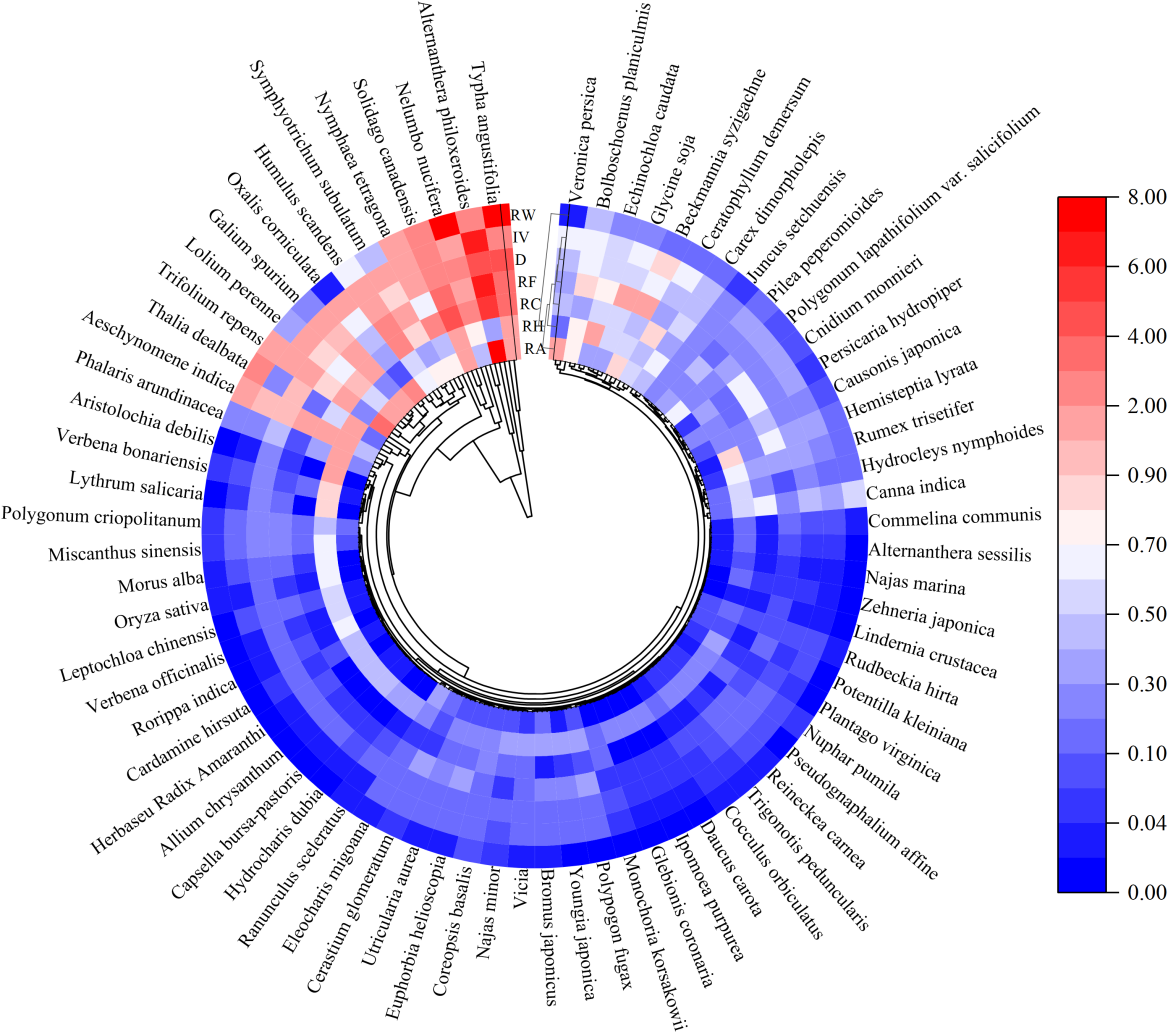
The phylogenetic tree (n=220 species) was derived from the sequence number of species in *Flora of Anhui Province*. Cells from the inside to the outside of the phylogeny are plant parameters: RA, relative abundance; RH, relative height; RC, relative coverage; RF, relative frequency; D, dominance; IV, important value; and RW, relative weight.

A total of 220 vascular plants from 64 families and 168 genera were identified in 19 restored wetlands around Chaohu Lakeside (**Figure 2**). The proportion of local species in the regional species pool varied among different plant taxa. Among them, the species from Poaceae, Asteraceae, Cyperaceae, Polygonaceae, and Fabaceae contributed to15%, 10.91%, 5.91%, 5.45%, and 4.55% of the total species, respectively. Poaceae, particularly annual and biennial weeds, were more dominant than forbs in wetlands, that embraced 33% of richness and 38.76% of abundance (Sutherland, 2004). Poaceae, Cyperaceae, and Polygonaceae were major contributors to the weed list in this region, characterized by fast growth rate, high adaptability, and rapid reproduction (Baker, 1974).

Annual species primarily propagated through seeds, while most perennial species spread through asexual reproduction. The total mass of annual species’ seeds was twice as much as that of perennial plants in the soil of emergent communities (Drahota and Reichart, 2015). Aquatic vegetation restoration provided high-energy food sources (seeds, tubers, and submerged aquatic plants) tend to enhance waterfowl diversity of wetlands (Lishawa et al., 2020; Zhou et al., 2020). Species like *Beckmannia syzigachne* (*B. syzigachne*) and *Echinochloa caudata* (*E. caudata*), prevalent in abandoned farmland, were often used as pioneer species for wetland restoration (Yan et al., 2023). However, perennial species such as *S. canadensis*, *P. australis*, and *T. angustifolia* are not edible by birds, and their proliferation can reduce bird diversity in restored wetlands. This condition can be revised by controlling invasive plants (Lishawa et al., 2020).

The predominant species of upland vegetation formation was mainly originated from the Asteraceae and Fabaceae families, such as *Solidago canadensis* (*S. canadensis*), *Erigeron canadensis* (*E. canadensis*), *Sonchus oleraceus* (*S. oleraceus*)*, Glycine soja* (*G*. *soja*) and *Vicia sativa* L (*V. sativa*)*. S. canadensis* was initially introduced as an ornamental plant, but escaped and became pervasive in wetlands (Wang et al., 2021). The shading tolerance capacity of *S. canadensis* enabled it adapted to various environmental variables (Wu et al., 2020). On the other hand, *G. soja*, a native species under state protection (category II), is known for its climbing and strangling behavior towards other species. While Cyperaceae and Polygonaceae were mainly obligate or facultative wetland species like *Bolboschoenus planiculmis*, *Cyperus difformis*, *Persicaria lapathifolia*, and *Persicaria orientalis* which mainly distributed in wet grassland and emergent vegetation formations.

According to the plant source, the proportion of native species, artificially planted species, alien species, and alien invasive species accounted for 63.64%, 21.82%, 3.18%, and 11.36%, respectively. The top five native species with higher important value were *Phragmites australis* (*P. australis*), *Paspalum distichum* (*P. distichum*), *Cynodon dactylon* (*C. dactylon*), *Potamogeton crispus* (*P. crispus*), and *T. angustifolia*, with IV of 0.054, 0.043, 0.042, 0.030, and 0.026 respectively. Among many invasive plants, *A. philoxeroides, S. canadensis*, and *E. canadensis* tended to be more pervasive and predominant in lakeside wetlands with IV values of 0.064, 0.029, and 0.026, respectively. *A. philoxeroides*, as an invasive species, can spread quickly through vegetative propagation and adapt well to diverse habitats under different light regimes conditions (Pan et al., 2006). *S. canadensis* exhibits high drought stress tolerance, low nutrient stress tolerance, high fecundity, and diffusion ability, making it the most threatened invasive species in wetlands (Wang et al., 2023). Another invasive species *Erigeron canadensis* (*E. canadensis*) with high growth rate that can secrete allelochemicals to inhibit the growth of neighboring plants (Yan et al., 2020).

### 3.2 Interspecific correlation of plants

**Figure 3:**
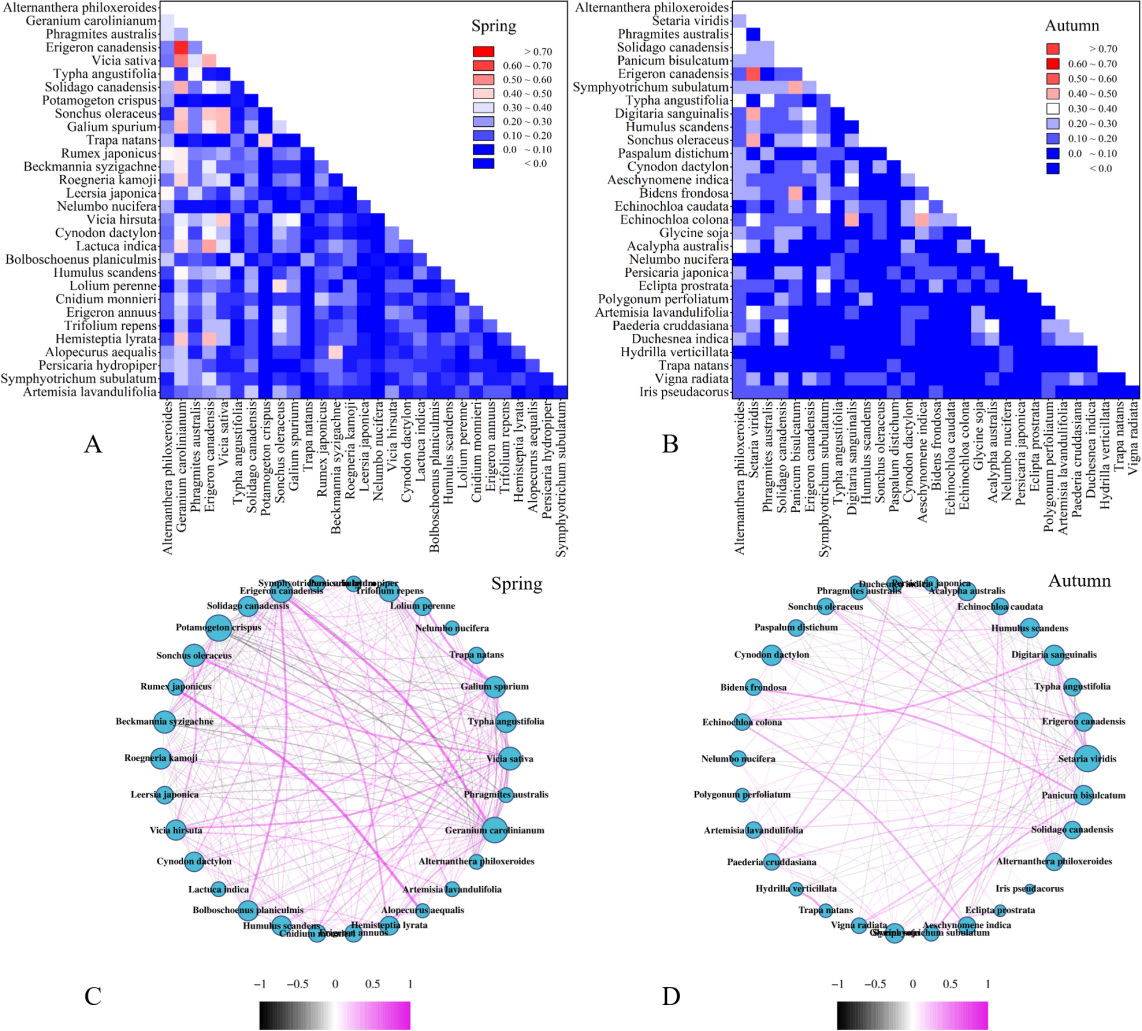
Interspecific relationship among the top thirty predominant species in the regional species pool of the Chaohu lakeside wetlands. **(A, B)** Ochiai index. From up to bottom and left to right, species quoted by its frequency from high to low. **(C, D)** Spearman rank correlation coefficient (r_ij_) of species in the spring and autumn, respectively. The purple curve represents positive correlation while the black curve represents negative correlation. The absolute value of r_ij_ under 0.1 were neglected.

To determine interspecific relationship among vascular plants, a total of 30 predominant species and 435 pair of relation were analyzed in the spring and autumn, respectively (**Figure 3**). In spring, alien invasive plants such as *A. philoxeroides, G. carolinianum*, *E. canadensis*, and *S. canadensis* exhibited higher dominance. *A. philoxeroides*, as semiaquatic species showed a positive correlation with hydrophyte and macrophyte like *Rumex japonicus* (*R. japonicus*), *Leersia japonica*, and *T. angustifolia* with OI exceed 0.35. Conversely, *A. philoxeroides* exhibited a negative correlation with xerophytes and mesophytes like *G. carolinianum*, *E. canadensis*, and *S. oleraceu*, with OI value of 0.31, 0.07, and 0.03, respectively. On the other hand, *G. carolinianum, E. canadensis*, and *S. canadensis* are xerophilous or mesic plants mainly found in upland vegetation formation. Furthermore, the *G. carolinianum* and *E. canadensis* showed positively correlated with most xerophilous or mesic species in spring but negative correlations in autumn. Specifically, *S. canadensis* exhibited a positive correlation with vine and herbaceous plants with larger size while it obtained a negative correlation with herbaceous plants with smaller size.

The dominant native species identified in this study were *P. australis, Vicia sativa*, and *T. angustifolia.* Among them, *P. australis* showed a positive interspecific correlation with *T. angustifolia*, with an OI of 0.34 and χ^2^ of 13.64, indicating a significant overlap in their ecological niche. Both *P. australis* and *T. angustifolia*, known as obligate wetland species, are widely utilized in wetland restoration projects. The positive correlation observed in invasive plants suggested their superior competitive ability over native species. Furthermore, the intensity of interspecific competition between invasive and native plants was more intense than that among different invasive species. Interestingly, xerophilous or mesic species such as *Humulus scandens* (*H. scandens*), *Nelumbo nucifera* Gaertn (*N. nucifera*) and *Hydrilla verticillate* (*H. verticillate*) showed no correlation with macrophytes, for that most macrophyte were monocultured. Additionally, the presence of hydrophytic habitats have effectively restricted the spread of xerophilous and mesic invaders (Dou et al., 2022).

### 3.3 Niche partition of plants

**Figure 4:**
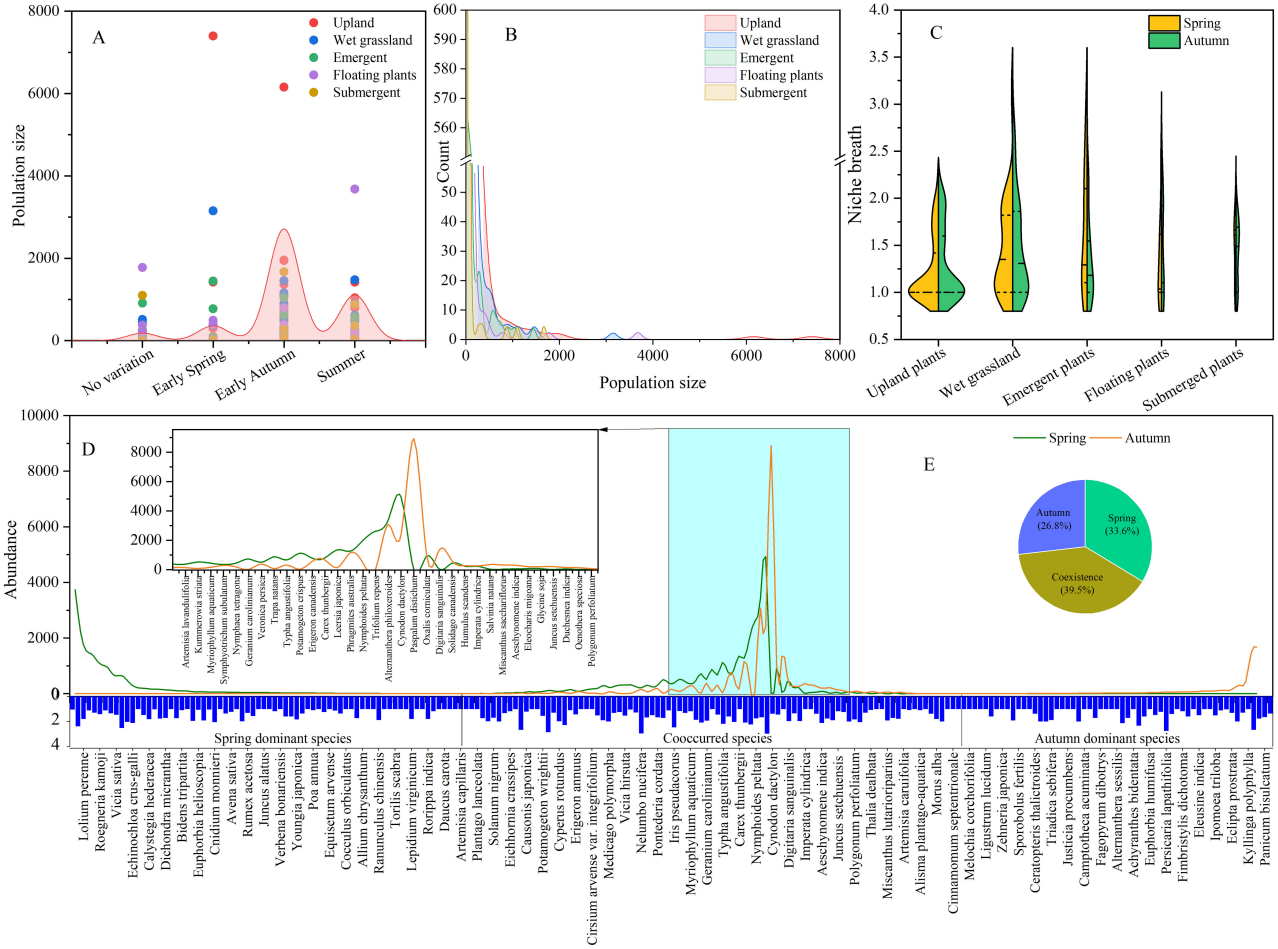
The temporal and structural niche of 220 vascular plants among 19 lakeside wetlands (**(A)** plants phenology; **(B)** population size of species; **(C)** spatial niche; **(D)** temporal niche; **(E)** partitioning of temporal niche).

The strategies employed by plants to alleviate competition involves niche partitioning in both space and time (**Figure 4**). The distribution of vascular plants within various vegetation formations such as upland plants, wet grassland plants, emergent plants, floating-leaved plants, and submerged plants was 51.36%, 31.36%, 8.64%, 4.55%, and 4.09%, respectively (Yang et al., 2024). As shown in **Figure 4C**, the average niche breadth upland plants, wet grassland plants, emergent plants, floating-leaved plants, and submerged plants were 1.28 ± 0.36, 1.53 ± 0.52, 1.67 ± 0.60, 1.48 ± 0.52, and 1.58 ± 0.49, respectively. This result supported the hypothesis that the niche of functional groups in wetlands was predominantly influenced by hydrological conditions (Deane et al., 2017). Meanwhile, species of wet grassland and emergent vegetation formations usually obtained higher tolerance to hydrological changes. Specifically, species like *A. philoxeroides*, *N. nucifera*, and *Rumex japonicus* exhibited wider niche breadth exceeding 2.85 compared with *S. canadensis* of 1.34. Due to the coexistence of these adaptive species that contributed to the ecotone’s higher biodiversity (Yang et al., 2024).

Another factor that influences species fluctuations is the annual climate variability. A previous study by Zheng et al. (2024) highlighted the significant impact of temporal shifts and functional traits on grassland productivity. In contrast, Usinowicz et al. (2017) found that the benefits of asynchronous coexistence were more pronounced in tropical and subtropical forests compared to temperate forests. **Figure 4D** illustrates the explicit partition of plants’ temporal niche. The result indicated that 160 species were observed in spring and 148 species in autumn. The distribution of species along the X-axis indicates that 33.6% of investigated species occurred in spring, 26.8% in autumn, and 39.5% in both seasons. Wang et al. (2012) noted that 25.45% of species matured from late spring to summer, with 8.18% achieving the highest biomass in early spring and 4.09% maintaining consistent biomass throughout the year. Additionally, 62.27% of plants matured in early autumn (**Figure 4A**). The partition of plants reproductive phenology can effectively enrich the seed bank of wetland as well as facilitate the survive rate of plants.

According to the reproductive capacity of plants, annual species generally embraced shorter germination times compared to perennial species (Shipley and Parent, 1991). In lakeside wetlands, the species pool consisted of 97 annual plants (44.09%) and 123 perennial herbs (55.91%). Spring-dominant species were typically annual herbs with smaller size and biomass, such as *Azolla pinnata*, *Spirodela polyrhiza*, and *Galium* sp*urium*. Annual herbs tend to spread quickly due to shorter generation times or higher fecundity, giving them a selective advantage over biennials and perennials (Sutherland, 2004). On the other hand, perennial hygrophytes like Phragmites, Typhaceae and Cyperaceae embrace well-developed rhizomes or corms that provide flooding tolerance (Wang et al., 2022). In the initial stages of wetland restoration, annuals typically dominate the species composition, but there is a gradual shift towards perennials in long-term succession (Jenkins et al., 2021).

Species predominant in autumn were mainly from the Poaceae family, such as *Echinochloa colona*, *E. caudata*, and *Setaria viridis*, with seeds maturing in autumn. These species were often accompanied by planted species like *N. nucifera*, *Nymphaea tetragona*, and *T. angustifolia*. Notably, *G. carolinianum* matured in spring and completed its life cycle in summer. While most species withered in autumn, *Panicum bisulcatum* bloomed and outperformed others in terms of height and coverage. Species coexisting in spring and autumn were characterized by perennial macrophytes with greater competitiveness in terms of height and biomass. Additionally, species with wider temporal niche tended to possess higher niche breadth as well (**Figure 4D**).

### 3.4 Traits tradeoff of plants

Ultimately, the interspecific competition among plants is a competition for resources. The competitiveness of plants is usually size asymmetric that the larger plants typically receiving a greater share of light resources which may suppress neighboring plants’ fitness (Aschehoug et al., 2016). In this study, relative height (RH) and relative coverage (RC) were introduced into the Lotka-Volterra model to assess the competitiveness and strategies for plant coexistence in a large community (Silvertown, 2004; Tilman, 1990). Because majority (62.27%) of plants in this region reached maturity in the autumn, leading to the use of autumnal traits to determine stability conditions, while variations of traits between spring and autumn were used to assess the strategies of plants (Chesson, 2000).

**Figure 5:**
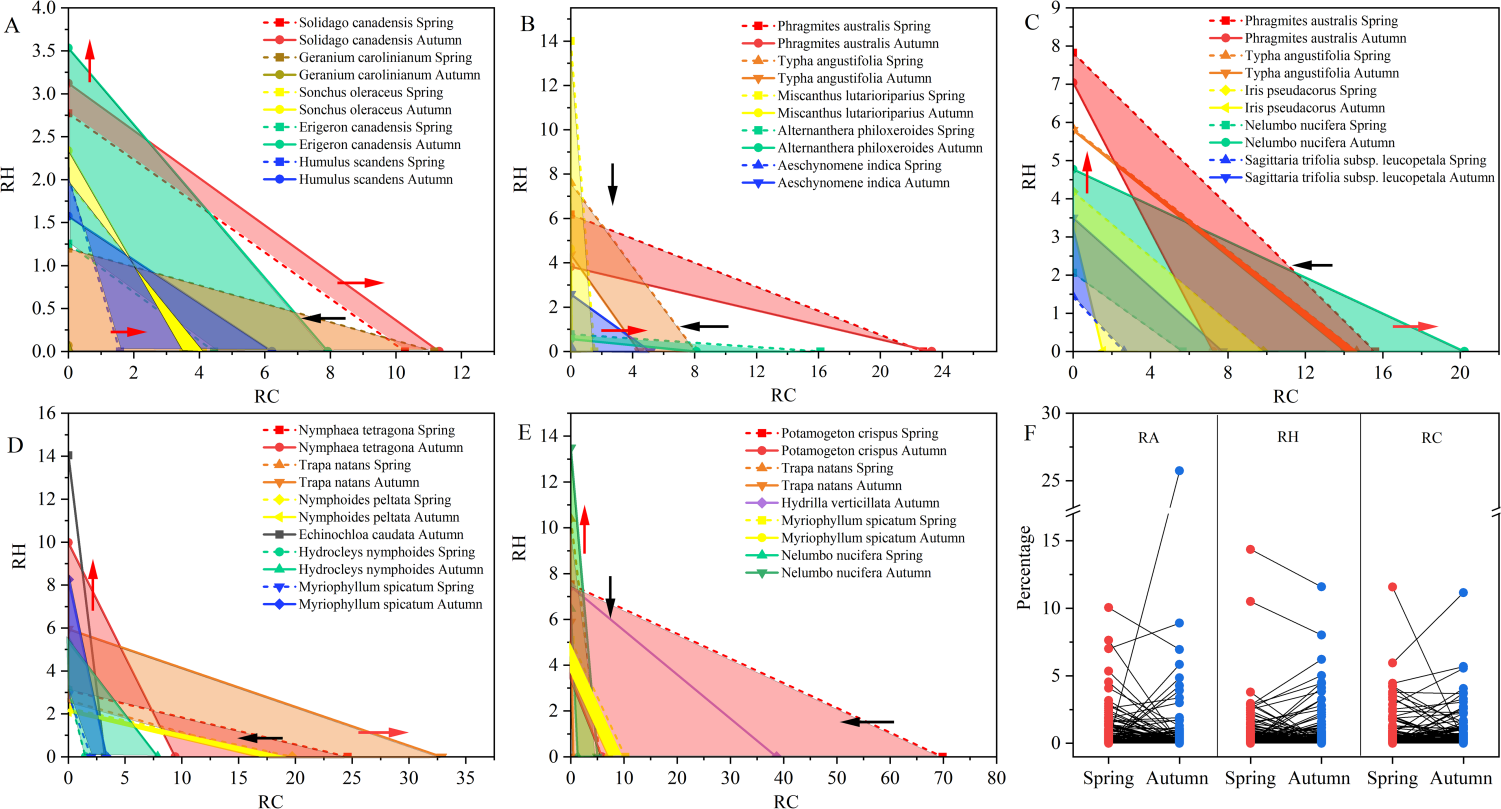
Variation of plants functional traits in different vegetation formations: **(A)** upland vegetation formation; **(B)** wet grassland vegetation; **(C)** emergent plants; **(D)** floating-leaved plants; **(E)** submerged plants; **(F)** seasonal change of plants’ traits; RA, relative abundance; RH, relative height; RC, relative coverage; The red arrows mean the expansion of traits while the black arrows represent the shrink of traits.

The coverage and height tradeoff are the most common strategies for plant coexistence in the presence of interspecific competition. If $\frac{\Delta RH}{\Delta RC}>1$ , the species is determined by the height tradeoff strategy. If $\frac{\Delta RH}{\Delta RC}<1$ , the species is determined by the coverage tradeoff strategy. When $\frac{\Delta RH}{\Delta RC}\approx 1$ , the species is determined by both coverage and height tradeoff strategies. When the $\frac{\Delta RH}{\Delta RC}$ is negative, the equation is substituted by $\frac{RH}{RC}$ . Plants applied height tradeoff strategy are these species like *E. canadensis*, *Miscanthus lutarioriparius*, *E. caudata*, and *N. nucifera*, while the coverage tradeoff strategy is observed in *G. carolinianum*, *A. philoxeroides*, *Trapa natans*, and *P. crispus* (**Figure 5**).

Our results indicated that plants in different vegetation formations may adopt disparate strategies. In upland vegetation formation, *S. canadensis* was predominant by height and coverage while *E. canadensis* and *G. carolinianum* were driven by height and coverage strategies, respectively. Additionally, plants in different habitat may also adopt different strategies. For example, *P. australis* adopted coverage strategy in hygrophilous habitat while it employed height strategy in aquatic habitat. According to the Lotka-Volterra model, species with trading strategies tend to coexist, while non-trading strategy species are more likely to be excluded by competitive species (De Mazancourt and Schwartz, 2010). The weaker competitors increase their fitness via traits trade off while stronger competitors are suppressed by their conspecific neighbors (Stoll and Prati, 2001). The increase in dominance of a species in one resource comes at the expense of competitiveness in another resource.

## 4 Discussion

**Figure 6:**
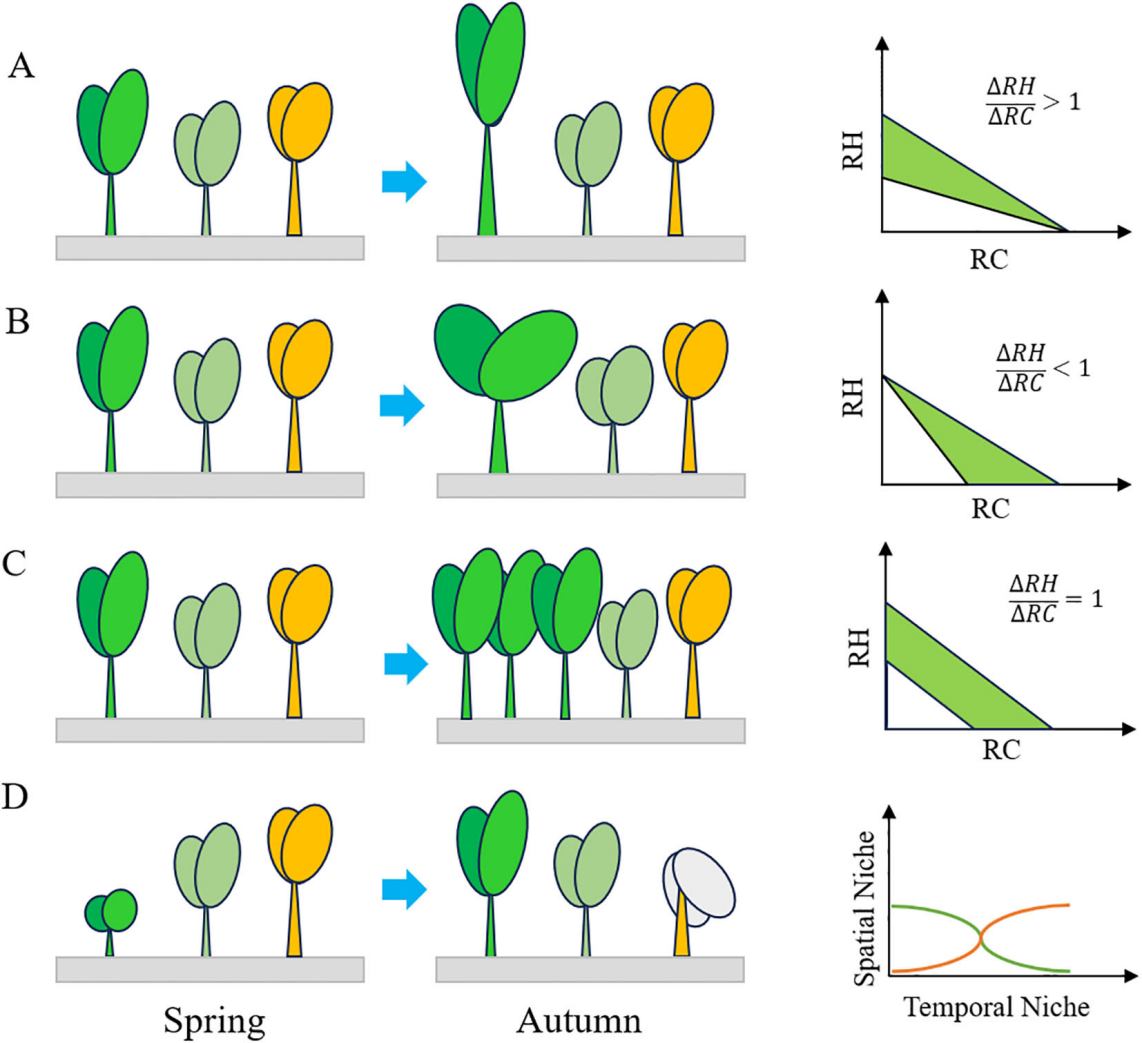
The strategies of plants coexistence under competition: **(A)** height tradeoff strategy; **(B)** coverage tradeoff strategy; **(C)** height and coverage tradeoff strategy; **(D)** niche partitioning strategy; RH, relative height; RC, relative coverage; Δ*RH*, variation of relative height; Δ*RC*, variation of relative coverage.

Plant strategies in coping with competition can be summed up as spatial and temporal niche partitioning and trait tradeoffs (**Figure 6**). Temporal niche differentiation is primarily achieved through phenology partitioning, which reduces competition intensity and promotes species coexistence (Pak et al., 2023). As the resources including light, water, soil nutrient availability variate during the year, the partitioning of plant phenology allowing competitors to coexist (Chesson, 1994). Short-lived species tend to complete their life cycles under favorable environmental conditions to avoid dreadful environment (Hereford et al., 2017). Our findings revealed that 33.6% of species occurred in spring, 26.8% in autumn, and 39.5% in both seasons. Specifically, 25.45% of species matured in early summer, while 62.27% matured in autumn. Species that matured in early spring were typically small-sized, such as *Vicia hirsuta* and *Medicago polymorpha*, with lower competition, leading to variations in plant maturity timing. Notably, the endangered fern *Ceratopteris chingii* completed its life cycle in autumn (Guan et al., 2019). Special attention should be given to these annual species with specific and narrower temporal niches.

Among the five vegetation formations of restored wetlands, emergent plants had the widest niche breadth of 1.67 ± 0.60, while upland vegetation species had the lowest niche breadth of 1.28 ± 0.36. The emergent plant vegetation formation was predominantly composed of facultative species that can survive in both upland and wet grassland of wetlands. On the other hand, upland vegetation was dominated by xerophyte and mesophyte species, resulting in higher plant richness of 81 species but a lower niche breadth of 1.28. There was no correlation between upland species and most hygrophytes, indicating that habitat heterogeneity promoted vegetation partitioning. The vegetation formations in wetlands were primarily influenced by soil moisture, with the wetland hydrology regime playing a key role in regulating plant communities (Regmi et al., 2021). In particular, the inundation time of wetlands was found to influence species niche differentiation in wetlands (Foti et al., 2012). By managing the hydrological regime of seasonal flooding lakeside wetlands, it was possible to control the spread of xerophytic invasive species such as *G. carolinianum, E. canadensis*, and *S. canadensis.*

Traits tradeoff in height and coverage were primary strategies of plants to acquire more resources under interspecific competition. The established species like *P. australis*, *T. angustifolia*, and *S. canadensis* are usually taller in the communities, while subordinate species inclined to pursue for a height tradeoff to compete for light resources when there was little height difference. Less competitive species turned to pursue for a coverage tradeoff, investing more nutrients in their leaves to maximize light absorption. For instance, plants with high specific leaf area tended to occupy areas with higher resources, whereas those with lower specific leaf area tended to establish in areas with lower resources (Adler et al., 2013). Plants adopting coverage tradeoff strategy are prostrate plants like *Trifolium repens*, *H. scandens*, *Kummerowia striata*, *Actinostemma tenerum*, *Causonis japonica* which incline to expand their coverage when competed with other species (Eskelinen et al., 2022). However, the pervasive of these species may lead to the exclusion of other species due to light shading effects. Another way for plants to alleviate interspecific competition is the tradeoff height and coverage strategies. This strategy was mainly achieved through the increase in individual numbers, which could, in turn, intensify intraspecific competition. Species commonly found in this scenario are most Cyperaceae, Poaceae, and Juncus species like *Carex brachyathera*, *Cynodon dactylon*, *P. distichum*, and *Juncus effuses*.

To recover and maintain plant diversity in restored wetlands, wetland conservators can adopt the following strategies: Firstly, protect annual species and control perennial plants to ensure the sustainability of annual species propagule. Secondly, clear invasive species or replace them with native plants which have highly competitive capacities that can coexist with alien invasive plants through height, coverage tradeoff strategy, or both of it. Thirdly, establish a biological defense line by safeguarding the hydrology regime and reducing the diffusion risk of non-native species (Deane et al., 2017; Raulings et al., 2010). In general, priority should be given to plant functional traits, landscape configuration, and hydrology regime in the restoration and maintenance of wetlands.

## 5 Conclusion

To investigate the coexistence mechanisms and strategies of plants under interspecific competition, a comprehensive study was conducted among 220 vascular plants from 19 restored wetlands. The OI index indicated that 62.41% of species pairs embraced a negative correlation while 37.59% species pairs were positive. Notably, invasive species such as *A. philoxeroides*, *G. carolinianum*, *S. canadensis*, and *E. canadensis*, along with native species like *V. sativa* and *S. viridis*, embraced higher competitiveness than neighboring species. The primary strategies for different plant coexisted including spatial and temporal niche partitioning, as well as tradeoffs in height and coverage. Emergent plants obtained a wider niche breadth of 1.67 ± 0.60 compared to upland species with the narrowest niche of 1.28 ± 0.36. In temporal scale, species occurred in spring, autumn, and both spring and autumn reached 33.6%, 26.8% and 39.5%, respectively. Phenology character of plants indicated that the proportion of species matured in spring, summer and autumn accounted for 8.18%, 25.45% and 62.27%, respectively. Height tradeoff strategies were predominantly observed in communities of *P. australis*, *T. angustifolia*, and *S. canadensis*, while coverage tradeoff strategies were more prevalent in *Trifolium repens*, *H. scandens*, and *Kummerowia striata* communities. Overall, the coexistence strategies including spatial and temporal niche partitioning, as well as tradeoffs in height and coverage traits among vascular plants that hold promise for biodiversity conservation and the management of alien invasive species.

## Data Availability

The datasets presented in this study can be found in online repositories. The names of the repository/repositories and accession number(s) can be found below: https://datadryad.org/stash/share/hlC-twvPymbqGh2wsUbZwp6tu6TBAbTtTjodHjSo5tc.
