# Supplementary material for: Niche partitioning and trait tradeoff strategies enable plants to coexist under interspecific competition in restored wetlands
Source: Front Plant Sci. 2025 Apr 2;16:1539136. doi: 10.3389/fpls.2025.1539136 (PMC12000035; doi:10.3389/fpls.2025.1539136)
Supplement: Supplementary file 1 [file Table1.docx]

**Supplementary Tables and Figures.**

**Niche partitioning and trait tradeoff strategies enable plants to coexist under interspecific competition in restored wetlands**

Shenglin Yang ^1,2,3,^ ^†^, Zhen Yuan ^1,2,^ ^†^, Bibi Ye ^1,2*^, Feng Zhu^1,2,3^, Xiaoxian Tang^4^, Rui Gao^4^, Zhaosheng Chu ^1,2,3*^, Xiaowei Liu ^5*^

1.National Engineering Laboratory for Lake Pollution Control and Ecological Restoration, State Key Laboratory of Environmental Criteria and Risk Assessment, Chinese Research Academy of Environmental Sciences, Beijing 100012, China

2.State Environmental Protection Key Laboratory for Lake Pollution Control, State Environmental Protection Key Laboratory of Drinking Water Source Protection, Chinese Research Academy of Environmental Sciences, Beijing, 100012, China

3. College of water science, Beijing Normal university, Beijing 100875, China

4. Institute of Lake Ecology and Environment, Anhui Provincial Lake Chaohu Administration, Hefei, 230000, China

5. School of Biology, food and Environment, Hefei University, Hefei 230601, China

The completion date of studied19 restored wetlands in Chaohu lakeside are listed in **Table S1.**

**Table S1.** The completion date of 19 restored wetlands.

|  | Wetlands | Restoration Projects |  | Complete time |
| --- | --- | --- | --- | --- |
| 1 | SBLW(Ⅰ) | Changlinhe Provincial level Wetland Park | Returning farmland to wetland; Dredging, Reforestation; Aquatic plants recover | 2022 |
| 2 | SBLW(Ⅱ) |  | Returning farmland to wetland; Habitat restoration; Artificial ponds; Aquatic plants recover | 2021 |
| 3 | YDH | Yudaihe Municipal Wetland Park | Returning farmland and fishpond to wetland; Aquatic plants recover | 2020 |
| 4 | HTH | Chaohu Bandao National Wetland Park | Returning fishpond to wetland; Expanding of surface-flow wetland; Wetland Plants recover | 2019 |
| 5 | LX |  | Returning farmland and fishpond to wetland; | 2022 |
| 6 | TY |  | Returning farmland and fishpond to wetland; Artificial ponds; Wetland Plants recover; | 2021 |
| 7 | SC | Zhegaohe Provincial level Wetland Park | Returning fishpond to wetland; Artificial ponds; Aquatic plants recover; | 2016 |
| 8 | FXH | Binghu National Wetland Park | Habitat restoration; Reforestation; Aquatic plants recover | 2022 |
| 9 | BH |  | Dredging; Damming; Habitat restoration; Reforestation; Aquatic plants recover | 2022 |
| 10 | PH | Paihe Municipal Wetland Park | Returning fishpond to wetland; Dredging; Aquatic plants recover | 2022 |
| 11 | XFW | Paihe Municipal Wetland Park | Reforestation | 2022 |
| 12 | DAW |  | Returning farmland to wetland; Artificial ponds; Wetland Plants recover; | 2021 |
| 13 | QFZ | Qifengzhou Provincial level Wetland Park | Returning farmland to wetland; Reforestation; Connecting water system; | 2021 |
| 14 | YLW |  | Returning farmland to wetland; Connecting water system; Aquatic plants recover | 2021 |
| 15 | GS | Maweihe National Wetland Park | Returning farmland to wetland; Aquatic plants recover; | 2021 |
| 16 | CTLW |  | Returning farmland to wetland; Aquatic plants recover; | 2021 |
| 17 | ZH | Huailin Provincial level Wetland Park | Returning farmland and residential area to wetland; Artificial ponds; Wetland Plants recover; | 2021 |
| 18 | SCH |  | Reforestation; Slope protection; invasive plants elimination | 2021 |
| 19 | GL |  | Reforestation, aquatic plants recover, invasive plants elimination | 2021 |

The description of ten functional traits of plant guilds is listed in **Table S2**.

**Table S2** The description of functional traits of plants guilds in lakeside wetlands of Lake Chaohu.

| Number | Traits | Description | Values |
| --- | --- | --- | --- |
| 1 | Carbon storage | Capacity to store carbohydrate than can be broken down and allocated to new growth (thickened roots and stems, bulbs, rhizomes) | Using in dendrogram acquisition no specialized storage organs = 0; slightly storage organs = 1; strong storage organs = 2 |
| 2 | Carbon immobilization | Capacity to invest C in support tissue (xylem and bark), in compounds that cannot be broken down to be used in further bio-syhthesis | Using in dendrogram acquisition herbaceous monocots = 1; Herbaceous dicots = 2 |
| 3 | Life span | The period to complete its life cycle of plant, based on field observation | Using in dendrogram acquisition perennials = 1; annual or biennial = 2 |
| 4 | Life-forms | the relationship to the height of the water table, based on literatured3 and field observation | Using in dendrogram acquisition hygrophyte = 1; emergent = 2; floating = 3; submerged = 4 |
| 5 | Ramification | Degree of ramification at the base of the plant | Using in dendrogram acquisition no evident divided= 0; evident divided= 1; |
| 6 | Reproductive phenology | Seasonality of maximum production of flowers and fruits, based on literature3and field observation | Using in dendrogram acquisition no evident peak = 1; winter, autumn, early spring = 2; spring, late spring, spring–summer, late summer–early autumn =3; late spring–summer, summer = 4 |
| 7 | Percentage flowering of the year | Percentage flowering of the year, based on literature3and field observation | Using in dendrogram acquisition 0 –≤0.25% = 1; >0.25–≤0.50 = 2; 0.50–≤1= 3 |
| 8 | Maximum height | Maximum height of species above the ground with percent cover ≥20%, based on field observation | Using in dendrogram acquisition 0 –≤10 cm = 1; >10–≤50 = 2; 50–≤100 = 3; ≥100 = 4 |
| 9 | Leaf area | Leaf length ×width of per leaves (cm^2^)5, based on field observation | Using in dendrogram acquisition aphyllous = 0; >0–≤10 cm^2^ =1; 10–≤50 = 2; 50– ≤100 = 3; >100 = 4 |
| 10 | Edibility | The parts that can be feed for birds | Not feedable=0; Seed=1; Leaf =2; Stem=3; Fruit=4; |
